# “Do I meet the criteria for dementia…Really, where am I headed?”: Perspective of people with Parkinson's Disease on the diagnosis, evaluation and management of cognitive disorders

**DOI:** 10.1002/alz70858_103957

**Published:** 2025-12-26

**Authors:** Deepa Sriram, Dana Pourzinal, Daniel X Bailey, Deborah Brooks, Nadeeka N Dissanayaka

**Affiliations:** ^1^ The University of Queensland, Brisbane, QLD, Australia

## Abstract

**Background:**

Cognitive dysfunction is one of the most functionally impactful non‐motor symptoms of Parkinson's Disease (PD), resulting in lowered quality of life, earlier institutionalisation, and amplified financial burden. Dementia in PD is poorly recognised and managed, creating a significant unmet need for more appropriate diagnostic and post‐diagnostic care pathways. However, there are no best practice guidelines for cognitive evaluation and dementia diagnosis in PD. This study aimed to explore the perception and experiences of people living with PD regarding the diagnosis and management of their cognitive symptoms. This study was designed to inform a larger program of research aiming at developing best practice guidelines for cognitive evaluation in PD (PDCogniCare).

**Method:**

Seven qualitative online focus groups were conducted between May and August 2024 to gain an in‐depth understanding on participants' experiences of neuropsychological assessment, diagnosis, and post‐diagnostic support for cognitive impairment. Participants included people with PD with subjective cognitive decline (PD‐SCD, *n* = 6), mild cognitive impairment (PD‐MCI, *n* = 3), dementia (PDD, *n* = 4), and care partners (*n* = 2). Focus group recordings were transcribed verbatim and analysed using both deductive and inductive thematic analysis. The initial coding categories were based on the focus group discussion guide and formed the basis on which the themes were organised.

**Result:**

Four main categories with several inter‐related themes were identified: **Pre‐Assessment**: Medical professional Reluctance to Assess, Information to make Informed Choice, Opportunities to Detect and Refer for Assessment, Communication Lost in Translation; **Assessment**: Timing, Poor testing tool, Confronting and anxiety provoking; **Diagnosis**: Need for Transparency, Focused Discussion, Available Resources, Forward Planning; **Post Diagnostic Care**: Support person as advocates for person with dementia, Awareness on Risk Reduction, Telehealth.

**Conclusion:**

Findings highlight the unique clinical experience of people with cognitive impairment in PD. We identified critical gaps in the diagnosis and management of cognitive symptoms in PD clinics and their implications for people living with PD and their care partners. These results will be used to provide a lived experience perspective to a larger program of research (PDCogniCare, ultimately to improve the quality of life of people with PD through improved standards of care.

